# Multi-omics analysis reveals the interaction of gut microbiome and host microRNAs in ulcerative colitis

**DOI:** 10.1080/07853890.2023.2261477

**Published:** 2023-09-29

**Authors:** Lingyan Ma, Chenyang Hou, Hua Yang, Qu Chen, Wentao Lyu, Zhen Wang, Jianfeng Wang, Yingping Xiao

**Affiliations:** a State Key Laboratory for Managing Biotic and Chemical Threats to the Quality and Safety of Agro-products, Institute of Agro-product Safety and Nutrition, Zhejiang Academy of Agricultural Sciences, Hangzhou, China; b Greentown Agricultural Testing Technology Co., Ltd, Hangzhou, China; c Hangzhou Original Seed Farm, Hangzhou, China

**Keywords:** Colitis, gut microbiota, MicroRNA, metabolite, immune

## Abstract

**Background:**

Inflammatory bowel disease (IBD) is a chronic inflammation of the gastrointestinal tract that co-occurs with gut microbiota dysbiosis; however, its etiology remains unclear. MicroRNA (miRNA)-microbiome interactions play an essential role in host health and disease.

**Methods:**

To investigate the gut microbiome and host miRNA profiles in colitis, we used a Dextran Sulfate Sodium (DSS)-induced ulcerative colitis (UC) model. Metagenomic sequencing and metabolome profiling were performed to explore typical microbiota and metabolite signatures in colitis, whereas mRNA and miRNA sequencing were used to determine differentially expressed miRNAs and their target genes in the inflamed colon.

**Results:**

A total of 986 miRNAs were identified between the two groups, with 41 upregulated and 21 downregulated miRNAs in colitis mice compared to the control group. Notably, the target genes of these significantly altered miRNAs were primarily enriched in the immune and inflammation-related pathways. Second, LEfSe analysis revealed bacterial biomarkers distinguishing the two groups, with significantly higher levels of commonly encountered pathogens such as *Escherichia coli* and *Shigella flexneri* in the UC group, whereas beneficial species such as *Bifidobacterium pseudolongum* were more abundant in the control group. Microbiota metabolites histamine, N-acetylhistamine, and glycocholic acid were found to be downregulated in colitis mice. Spearman correlation further revealed the potential crosstalk between the microbiota profile and colonic miRNA, revealing the possibility of microbiome–miRNA interactions involved in IBD development.

**Conclusions:**

Our data reveal the relationships between multi-omic features during UC and suggest that targeting specific miRNAs may provide new avenues for the development of effective miRNA-based therapeutics.

## 1. Introduction

Inflammatory bowel disease (IBD) is a chronic inflammatory disorder of the gastrointestinal tract that comprises Crohn’s disease (CD) and ulcerative colitis (UC) [1,2]. UC is a chronic inflammatory disease that specifically targets the colon and can lead to the formation of ulcers and inflammation within the mucosal layer [3]. Patients grappling with UC commonly experience distressing symptoms, including abdominal pain, diarrhea, rectal bleeding, and significant weight loss [4,5]. Although the precise etiology of UC remains unclear, it is hypothesized that the gut microbiota, a diverse collection of microorganisms that inhabit the gastrointestinal tract, may play a critical role in the development and progression of UC [6]. In individuals susceptible to IBD, abnormal microbial colonization of the gastrointestinal tract may be the origin of such dysregulation [7].

Emerging research underscores the pivotal role of microRNAs (miRNAs), small non-coding RNA molecules, in the pathogenesis of colitis [8]. Dysregulation of miRNAs affects various cellular processes, including inflammation, apoptosis, and tissue repair, all of which are important in the development and progression of colitis [9,10]. Notably, several miRNAs have emerged as promising biomarkers and therapeutic targets in the context of colitis. For instance, the upregulation of miR-155 within the colonic tissues of individuals afflicted with IBD strongly suggests its implication in the pathogenesis of colitis [11]. Furthermore, in murine models of experimental colitis, a notable downregulation in miR-122 expression has been observed, aligning with the concurrent decline in liver function often associated with the disease [12]. This compelling connection between colitis and miRNAs highlights the potential of miRNA-based approaches in the diagnosis and treatment of colitis.

Investigations have shed light on how the gut microbiota exerts a profound influence on the expression of miRNAs within the colon, culminating in the dysregulation of pivotal cellular processes, notably including inflammation, apoptosis, and tissue repair [13,14]. Conversely, miRNAs are capable of regulating genes that are involved in bacterial sensing and host-microbe interactions, indicating that bidirectional interactions between host cells and gut microbiota *via* miRNA which participate in shaping the gut microbiota [15]. The interplay between gut microbiota and miRNAs provides a novel perspective on the pathogenesis of colitis and may offer potential diagnostic and therapeutic targets.

In the present study, we employed a Dextran Sulfate Sodium (DSS)-induced colitis model to investigate the gut microbiome and host miRNA profiles in UC. Metagenomic and metabolome sequencing were performed to explore the typical microbiota and metabolite signatures of colitis. Additionally, mRNA and miRNA sequencing was used to determine the differentially expressed miRNAs and target genes in UC. The interactions between the gut microbiome and host miRNA further reveal the potential interactions between the microbiota profile and host miRNA in UC, hoping to provide a better understanding of the miRNA-microbiota cross-talk and to facilitate their application in IBD.

## 2. Material and methods

### 2.1. Sample collection

Male C57BL6J mice (22–25 g) were purchased from the China National Laboratory Animal Resource Center (Shanghai, China) and kept in a temperature-controlled room (22 ± 2 °C) under a 12 h dark − light cycle. All animal procedures were approved by the Institutional Animal Care and Use Committee of Zhejiang Academy of Agricultural Sciences. Following a week of acclimation, the mice were grouped based on their body weight to ensure uniform starting points for various trials (*n* = 8/group). Colitis was induced by administration of 3% DSS (MW:36 − 50 kDa, Yeasen Biotech Co., Ltd, Shanghai, China) in drinking water for 7 days followed by 3 days of recovery. Disease activity index (DAI) was evaluated to assess the severity of the colitis according to methods described previously [16]. The colon of each mouse was excised and measured. Fecal sample from each mouse was aseptically collected and stored at −80 °C for future analysis. All animal procedures were approved by the Institutional Animal Care and Use Committee of the Zhejiang Academy of Agricultural Sciences (2018ZAASLA20).

### 2.2. Metagenomic sequencing

Genomic DNA was isolated using an E. Z. N. A. DNA Kit (Omega, Norcross, GA, USA). Metagenomic analyses were performed using the Novaseq 6000 platform (Illumina, USA). Briefly, 1 μg of genomic DNA was fragmented using a Covaris S220 focused ultrasound machine (Woburn, MA, USA), and fragments approximately 450 bp in length were used to prepare a sequencing library. Raw reads were quality-controlled using Trimmomatic to remove adapter contamination and low-quality reads [17]. Then, using the BWA mem algorithm, the read information that passed quality control was mapped to the host mouse genome database. Reads that remove host genome contamination and low-quality data are referred to as clean data for further analysis.

### 2.3. mRNA and Small RNA sequencing

Small RNAs and mRNA were sequenced on the Illumina platform by LC Sciences (Hangzhou, China). Small RNA libraries were prepared using approximately 5 µg of total RNA according to the TruSeq Small RNA Sample Prep Kit (Illumina, San Diego, CA, USA). Libraries were sequenced using Illumina HiSeq 2500 at LC-BIO following the vendor’s recommended protocol. Raw reads were subjected to an in-house program, ACGT101-miR (LC Sciences, Houston, Texas, USA) to remove adapter dimers, junk, low complexity, common RNA families (rRNA, tRNA, snRNA, and snoRNA), and repeats. TargetScan (v5.0) and Miranda (v3.3a) were used to predict miRNA-binding sites. The GO terms of miRNAs and miRNA targets were further annotated. The mRNA sequencing analysis was performed as previously described [18].

### 2.4. Metabolomic analysis

UHPLC-MS/MS analyses were performed using a Vanquish UHPLC system (Thermo Fisher, Germany) coupled with an Orbitrap Q Exactive^TM^ HF mass spectrometer (Thermo Fisher, Germany) at Biozeron Co., Ltd. (Shanghai, China). Samples were injected onto a Hypesil Gold column (100 × 2.1 mm, 1.9 μm) using a 17-min linear gradient at a flow rate of 0.2 mL/min. The eluents for the positive polarity mode were eluent A (0.1% FA in Water) and B (methanol). The eluents for the negative polarity mode were eluent A (5 mM ammonium acetate, pH 9.0) and B (methanol). The solvent gradient was set as follows: 2% B, 1.5 min; 2–100% B, 12.0 min; 100% B, 14.0 min; 100–2% B, 14.1 min; 2% B, 17 min. Q Exactive^TM^ HF mass spectrometer was operated in positive/negative polarity mode with spray voltage of 3.2 kV, capillary temperature of 320 °C, sheath gas flow rate of 40 arb, and auxiliary gas flow rate of 10 arb. The raw data files generated by UHPLC-MS/MS were processed using Compound Discoverer 3.1 (CD3.1, Thermo Fisher) to perform peak alignment, peak picking, and quantitation for each metabolite. metabolites were annotated using the KEGG and HMDB databases.

### 2.5. Enzyme-linked immunosorbent assay (ELISA)

The inflammatory state of serum was assessed through the measurement of mouse LPS (CSB-E13066m, Cusabio, Wuhan, China), the levels of inflammatory cytokine assay such as mouse IL-6 (EK206/3, MultiSciences), TNF-α (EK282/4, MultiSciences), colonic myeloperoxidase (MPO) (CSB-E08723m, Cusabio, Wuhan, China), and Lipocalin-2 (Lcn2) (CSB-E09410m, Cusabio, Wuhan, China) levels were measured by an ELISA kit according to the manufacturer’s recommendations. For colonic samples, supernatants were appropriately diluted (20-fold) with the recommended reagent diluent as per the kit instructions. Measurements were taken using a microplate reader set to 450 nm, with necessary corrections at 570 nm. For fecal samples, supernatants were diluted (50-fold) using the recommended reagent diluent. Plates were read at 450 nm with a correction at 570 nm.

### 2.6. Quantitative Real-time PCR

Total colonic RNA was extracted by using TRIzol (Vazyme Biotech Co., Ltd) and subsequently purified through lithium chloride precipitation [19]. Real-time quantitative PCR (RT-PCR) was performed with 2 × ChamQ SYBR Color qPCR Master Mix (Vazyme Biotech Co., Ltd.) following the manufacturer’s protocols. β-actin was used as the housekeeping gene for colonic tissue. The quantification of mRNA levels was measured using the threshold cycle (2^−ΔΔCT^) method as described previously [20]. The primers are shown in Supplementary Table 1.

### 2.7. Data analysis

All data are expressed as means ± SEM, and a *p*-value < 0.05 was considered statistically significant. miRNA/metabolites/microbiota species with a p-value < 0.05, and fold change ≥ 2 or FC ≤ 0.5 were considered to be differential biomarkers. Volcano plots were used to filter miRNAs or mRNA of interest based on log2 (Fold Change) and log10 (p-value). Principal coordinate analysis (PCoA) was performed based on the distance matrix of the Bray-Curtis dissimilarity of the microbial community between samples. Spearman’s correlation analysis was performed to reveal the interactions between the gut microbiome and host microRNA based on Spearman’s correlation coefficient > 0.9 and p-value < 0.01. Network Visualization was conducted using Cytoscape and Gephi software.

## 3. Results

### 3.1. The typical disease phenotypes in UC

**Figure 1.**
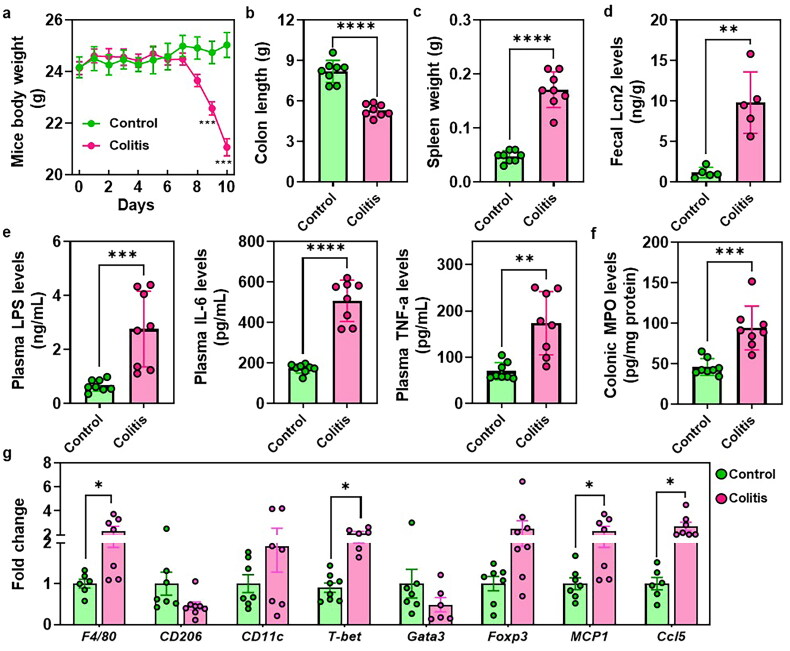
The typical disease phenotypes in UC. (a) Mice body weight. (b) Colon length. (c) Spleen weight. (d) Fecal levels of the inflammatory marker Lcn2. (e) Plasma LPS, IL-6 and TNF-α levels. (f) Colonic MPO levels. (g) RT-PCR results of the immune cells related genes expressions. *p < 0.05, **p < 0.01.

Figure 1(a) showed a significant reduction in mouse body weight and DAI score between days 7 and 10 in colitis mice when compared to the control groups (Figure 1(a) and Figure S1). Moreover, colitis-afflicted mice exhibited a pronounced reduction in colon length and a notable increase in spleen weight (Figure 1(b,c)). The elevated levels of fecal Lcn2, which serves as a broadly dynamic marker of gut inflammation was also found in the colitis mice (Figure 1(d)). Furthermore, the administration of DSS resulted in conspicuous elevations in plasma levels of LPS, IL-6, and TNF-α (Figure 1(e)). Additionally, colitis mice displayed a significant increase in colonic MPO level (Figure 1(f)). RT-PCR results revealed a substantial upregulation of immune-related genes, such as *F4/80*, *T-bet*, *MCP1*, and *Ccl5* in the colonic tissues of colitis mice, while *CD206* showed a tendency towards a decrease in the colon of colitis mice (Figure 1(g)).

### 3.2. Identification of differentially expressed mRNAs in UC

**Figure 2.**
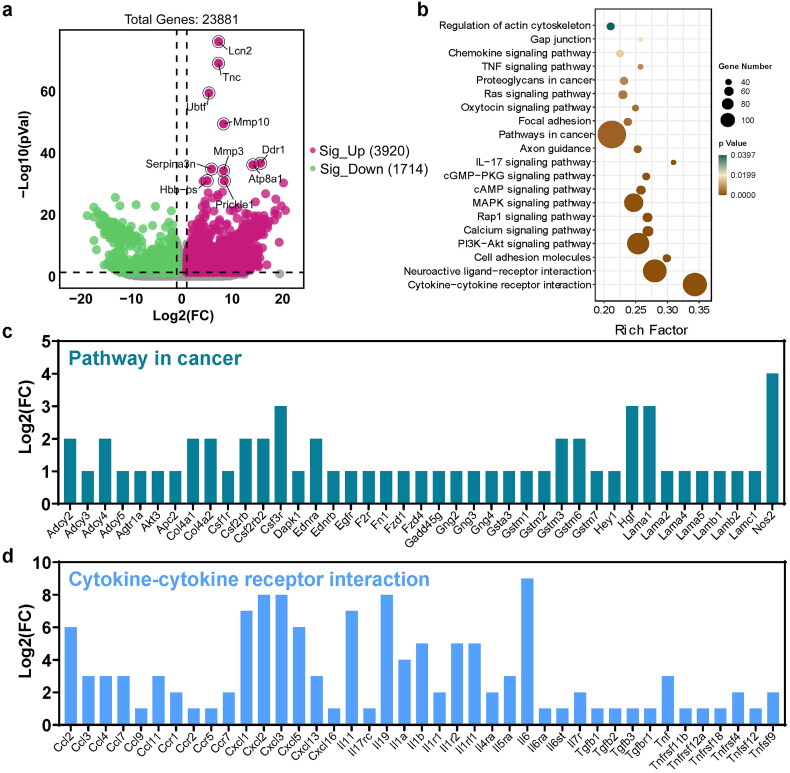
Identification of differentially expressed mRNAs in UC. (a) Volcano Plot shows the relationship between fold change and statistical significance of the differentially expressed mRNAs. The red points in the plot represent the up-regulated expressed mRNAs with statistical significance in colitis mice compared to the control group; the green points in the plot represent the down-regulated expressed mRNAs with statistical significance in colitis mice compared to the control group. (b) KEGG analysis of differentially expressed significant miRNAs in response to colitis. The top 20 of the most related parts were shown. The significant expressed genes involved in the ‘pathway in cancer’ (c) and ‘cytokine-cytokine receptor interaction’ (d).

To determine the differentially expressed mRNAs between colitis and normal tissues, RNA sequencing of colon tissues was performed. A total of 23881 genes were found to be differentially expressed, with 3920 genes upregulated and 1714 genes downregulated in colitis versus normal tissues. Functional enrichment analysis was performed on differentially expressed genes to predict their biological functions (Figure 2(a)). The results of the analysis showed that the differentially expressed genes were significantly enriched in pathways related to cancer, MAPK signaling pathway, PI3K-Akt signaling pathway, neuroactive ligand-receptor interaction, and cytokine-cytokine receptor interaction, according to KEGG pathway analysis (Figure 2(b)).

**Figure 3.**
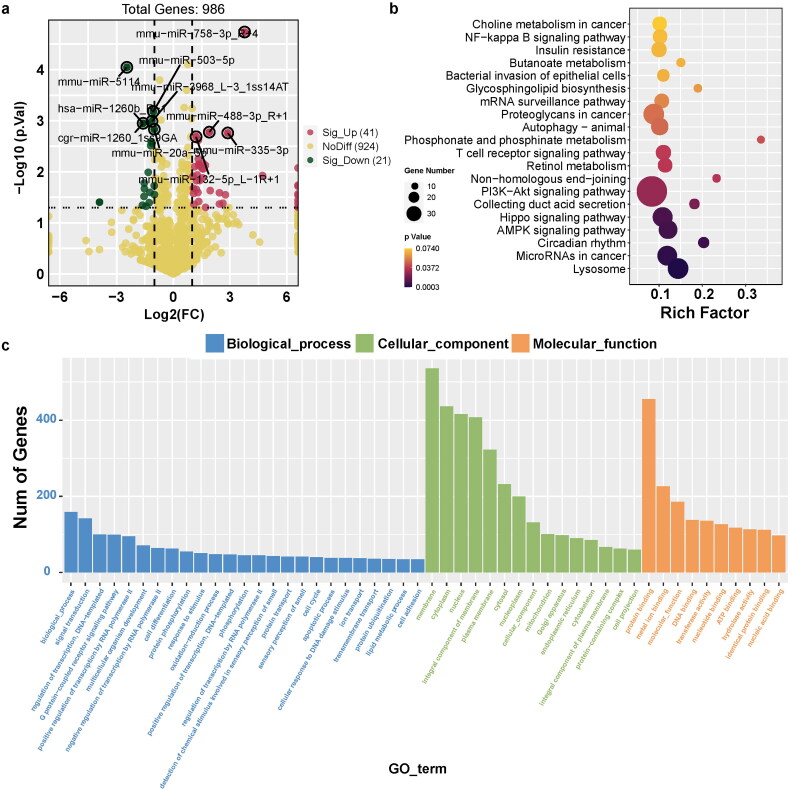
Identification of differentially expressed miRNAs and its-targeted genes in UC. (a) Volcano Plot shows the relationship between fold change and statistical significance of the differentially expressed miRNAs. The red points in the plot represent the up-regulated expressed miRNAs with statistical significance in colitis mice compared to the control group; the green points in the plot represent the down-regulated expressed miRNAs with statistical significance in colitis mice compared to the control group. (b) KEGG analysis of differentially expressed significant miRNAs in response to colitis. The top 20 of the most related parts were shown. (c) GO analysis of differentially expressed significant miRNAs in response to colitis. The most related parts were biological process, cellular component, and molecular function.

The most significant pathways, pathways in cancer, and cytokine-cytokine receptor interactions were further analyzed, with the associated genes shown in Figure 3(c,d), respectively (*p*-value < 0.05, fold change > 2). In comparison to normal tissues, *Nos2*, *Hgf*, *Lama1*, *Csf3r*, *Csf2rb2*, *Col4a1*, *Col4a2*, *Adcy2*, *Adcy4*, *Gstm3* and *Gstm6* were identified as the most significant genes in cancer pathways (Figure 2(c)). In Cytokine-cytokine receptor interactions, chemokines and their related receptors (e.g. *Ccl2*, *Cxcl1*, *Cxcl2*, *Cxcl3*), the interleukin family (e.g. *Il11*, *Il19*, *Il6*, *Il1b*), interferons (e.g. *Tnf*, *Tnfrsf4*), and transforming growth factor (e.g. *Tgfb1*, *Tgfb2*, *Tgfb3*) were the most enriched (Figure 2(d)).

### 3.3. Identification of differentially expressed miRNAs and its-targeted genes in UC

We detected 986 miRNAs of colon between the two groups, with 41 upregulated and 21 downregulated miRNAs compared to the control group (Figure 3(a)). Notably, the target genes of these significant miRNAs were primarily enriched in crucial pathways, such as the PI3K-Akt and Hippo signaling pathways, and miRNAs in cancer (Figure 3(b)). The GO function profiles revealed that the most significant GO functions were biological processes, including signal transduction and regulation of transcription (biological process); cellular components such as membrane, cytoplasm, and nucleus (cellular component); and molecular functions, such as protein binding, metal ion binding, and molecular function (Figure 3(c)).

**Figure 4.**
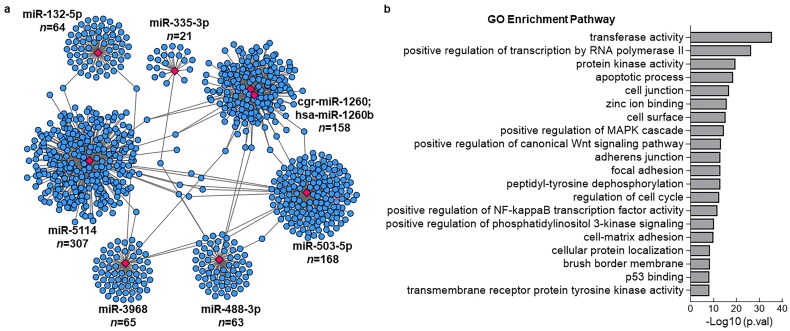
Visualization of identified miRNA with its associated target genes and GO analysis. (a) the Networks of identified most significant miRNAs (miR-758-3p, miR-503-3p, miR-5114, miR-1260b, miR-20a-5p, miR-132-5p, miR-488-3p, miR-3968, miR-503-5p, miR-335-3p) and their target genes with TargetScan_score > 90 and Miranda_energy < -20. (b) GO analysis of differentially expressed significant miRNAs in response to colitis. The top 20 of the most related parts were shown.

Among the upregulated miRNAs, miR-758-3p, miR-488-3p, miR-132-5p, and miR-335-3p were the most significant. Conversely, miR-5114, hsa-miR-1260b, miR-503-5p, miR-3968, cgr-miR-1260, and miR-20a-5p were the most significantly down-regulated miRNAs. We performed miRNA target prediction annotation to explore miRNA-mRNA interactions. Figure 4(a) shows a total of 1019 miRNA-target gene associations (edges) strongly associated with the most significant up- and downregulated miRNAs. The number of target genes related to each miRNA is shown in Figure 4(a). The GO analysis results suggested that the target genes were predominantly enriched in key biological processes, including transferase activity, positive regulation of transcription by RNA polymerase, protein kinase activity, apoptotic processes, and cell junctions (Figure 4(b)).

### 3.4. Differential analysis of microbiome profiles in UC

**Figure 5.**
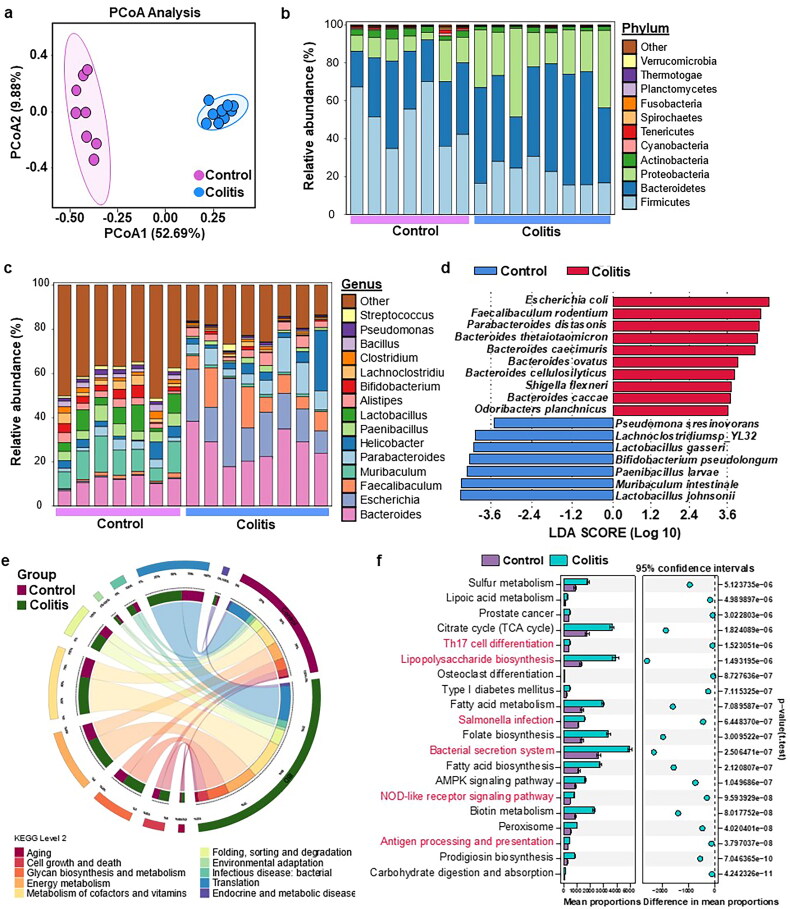
Differential analysis of microbiome profiles in UC. (a) Principal Coordinate analysis (PCoA) of microbiome profiles based on the out in the groups. Relative abundance of top 10 bacteria at the phylum (b) and genus levels (c). (d) LEfSe (linear discriminant analysis effect size) determines the bacteria species within two groups. (e) Circos plot showed a classified gut microbiota function of level 2. (d) The difference in gut microbiota function of the two-group based on stamp analysis.

Metagenomic sequencing of colonic content samples was employed to discern the differences in gut microbiota between the control and colitis mice. Principal coordinate analysis (PCoA) depicted in Figure 5(a) revealed a marked segregation of the bacterial communities between the two groups. (Figure 5a). At the phylum level, Bacteroidetes, Firmicutes, and Proteobacteria were dominant (Figure 5(b)). Notably, the UC group exhibited a significant increase in the relative abundance of Proteobacteria compared with the control group (Figure 5(b)). At the genus level, the UC group had higher levels of *Bacteroides*, *Escherichia*, and *Faecalibaculum*, whereas *Lactobacillus* and *Bifidobacterium* were more abundant in the control group (Figure 5c). Lefse analysis was performed to identify bacterial biomarkers that distinguished the two groups. The UC group had notably higher levels of commonly encountered pathogens, such as *Escherichia coli* and *Shigella flexneri*, while beneficial species, such as *Bifidobacterium pseudolongum* were more abundant in the control group.

KEGG pathway analysis revealed that glycan biosynthesis and metabolism, energy metabolism, and metabolism of cofactors and vitamins were the most enriched pathways at Level 2 (Figure 5(e)). At the KEGG level 3, the immune response and inflammation-related pathways, including Th17 cell differentiation, lipopolysaccharide biosynthesis, Salmonella infection, bacterial secretion system, NOD-like receptor signaling pathway, and antigen processing and presentation, were significantly upregulated in the UC group (Figure 5(f)).

### 3.5. Differential analysis of the microbiota metabolites in UC

**Figure 6.**
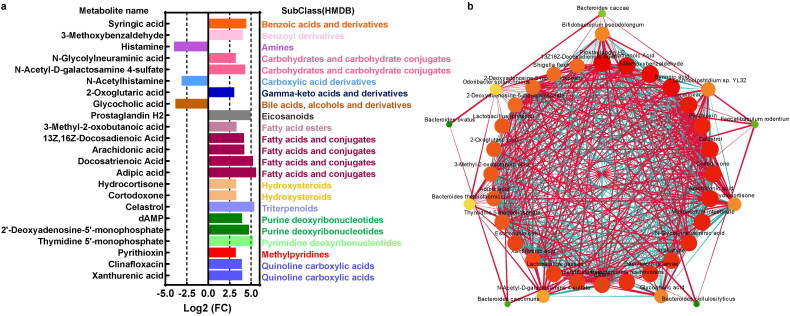
Differential analysis of the microbiota metabolites in UC. (a) Metabolites analysis of significant metabolites in colitis compared to the UC and its associated subclass (HMDB) based on the Log2 (fold change). (b) the interactions of bacteria species and metabolites with Spearman’ correlation coefficient > 0.9 and p-value < 0.01.

UHPLC-MS/MS analyses was used to detect and identify the metabolites in the intestinal microbiota. A total of 55 metabolites were found to be significantly regulated in the intestinal microbiota of colitis mice, and only 23 metabolites were annotated at the subclass (HMDB) level. As shown in Figure 6(a), three metabolites, including Histamine, N-Acetylhistamine, Glycocholic acid, were downregulated, while 20 metabolites, such as syringic acid, 3-Methoxybenzaldehyde, Adipic acid, Celastrol and Thymidine 5′-monophosphate, were upregulated in colitis mice (Figure 6(a)). Among these metabolites, most could be divided into fatty acids and conjugates (*n* = 5 metabolites), carbohydrate and carbohydrate conjugates (*n* = 2 metabolites), urine deoxyribonucleotides (*n* = 2 metabolites), urine deoxyribonucleotides (*n* = 2 metabolites), and quinoline carboxylic acids (*n* = 2 metabolites) (Figure 6(a)).

The network suggested interactions between gut microbiota and metabolites. For example, *E.coli* was significantly positively associated with clinafloxacin, celastrol, and cortodoxone, which were increased in the UC group (Figure 6b). In contrast, *Bifidobacterium pseudolongum* was found to be most significantly negatively related to xanthurenic acid, syringic acid, and clinafloxacin, but positively associated with histamine and N-acetylhistamine (Figure 6b).

### 3.6. The interactions of gut microbiome and host microRNA in UC

**Figure 7.**
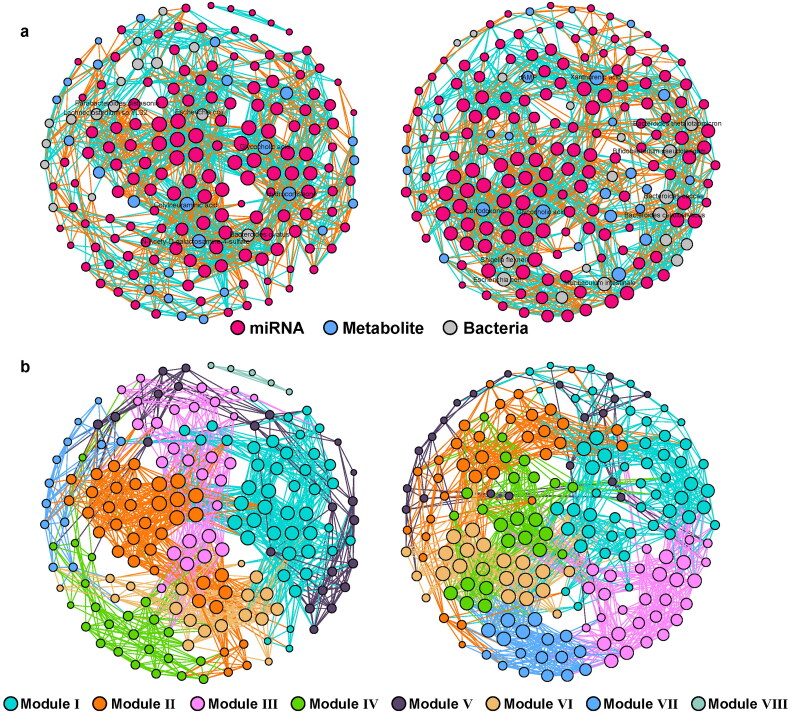
The interactions of gut microbiome and host microRNA in UC. The co-occurrence of gut microbiome profile including metabolites and bacteria species with host colonic miRNA in the groups clustered based on the types (a) and modularity (b). left: Control; right: UC.

Next, the co-occurrence of miRNAs, metabolites, and bacteria was performed using Spearman’s correlation (coefficient > 0.9, *p* < 0.01). As shown in Figure 7(a), there was a clear difference in the network structure between the UC and control groups. The results revealed more nodes and links in the UC network (195 nodes; 1641 edges; Supplementary Table 2) than in the control group (185 nodes; 1541 edges; Supplementary Table 2). Additionally, the control network contained eight main modules, while UC had only seven modules (Figure 7b; Supplementary Table 2). In the network of the control group, *Parabacterodides distasonis*, *Lachnoclostridium* sp.YL32, *E.coli* and *Bacteroides ovatus* showed the most significant association with the miRNAs, while Hydrocortiseone, Colylneuraminic acid, N-Acety-D-galactosamine-4-sulfate and Glycocholic acid were the main nodes of metabolites that were significantly associated with the miRNAs (Figure 7(a)). In the UC group, dAMP, cortodoxone, glycocholic acid, xanthurenic acid, *Bacteroides thetaiotaomicron*, *Bifidobacterium pseudolongum*, *Bacteroides caccae* and B*acteroides cellulosilyticus* were found to be significantly associated with miRNAs (Figure 7(a)). It is worth noting that microbiota metabolites seemed to show more co-occurrence with the miRNA in the control group, while in the UC group, Bacteroides related species also displayed more connections with miRNAs (Figure 7(a)).

## 4. Discussion

In the present study, we conducted multi-omics analyses of the gut microbiome and host miRNA profiles in UC to elucidate the crosstalk between the gut microbiota and host miRNA in colitis mice. The composition and diversity of gut microbiota play critical roles in the development of IBD [21]. In fact, the gut microbiota can undergo significant changes during the early stages of IBD, and fluctuations in microbiota composition are notably higher in IBD patients than in healthy individuals [22,23]. Specifically, IBD patients have significantly lower levels of beneficial bacteria, such as *Bifidobacterium longum*, *Eubacterium rectale*, and *Roseburia intestinalis* [24,25], whereas harmful bacteria, such as Bacteroides fragilis, exhibit an increased relative abundance and growth rate, further exacerbating the condition [26,27]. Consistent with these findings, our results demonstrated that the pathogenic bacteria E. coli and *S. flexneri* were enriched in colitis mice, whereas *B. pseudolongum* was significantly reduced.

We further focused on gut microbial metabolites as key regulators of their roles in the pathogenesis of IBD. We identified three paramount metabolites that exhibited significant alterations in mice afflicted with colitis as compared to their healthy counterparts. Among these metabolites, histamine and N-acetylhistamine assume central roles as major bioactive products within the histidine pathway, and they have been implicated in metabolic and immunological disorders [28]. Notably, previous investigations have highlighted the capacity of histamine to drive innate inflammation through the histamine 4 receptor in experimental murine colitis, underscoring its critical role in the pathogenesis of IBD [29]. However, it is intriguing to note that our study unveiled a reduction in histamine levels in colitis-affected mice, suggesting that microbiota-derived histamine may have distinct functions compared to its host-derived counterpart.

In addition, N-acetylhistamine was significantly increased in *in vivo* colitis models, suggesting its involvement in disease pathogenesis [30]. Additionally, bile acid signaling emerged as a critical regulator of various physiological processes, including inflammation [31]. Glycocholic acid (GCA), a crystalline bile acid, has been shown to have beneficial effects on gut mucosal damage and gastrointestinal inflammation [32]. In parallel with these findings, our study showcased a noteworthy decrease in GCA levels among colitis-afflicted mice, hinting at the potential therapeutic value of microbiota-derived metabolites in the context of IBD. In light of these findings, our research underscores the tantalizing prospect of targeting these pivotal microbial metabolites as a promising strategy for the treatment of IBD.

Recent research has shown growing interest in the role of miRNAs in the development of IBD. For example, miR-146a is upregulated in the inflamed mucosa of patients and is thought to play a role in regulating the innate immune response [33,34]. Similarly, miR-155 is upregulated in both UC and CD and is involved in the regulation of T cell activation [35]. Other miRNAs implicated in IBD development include miR-21, which is upregulated in the inflamed mucosa of IBD patients and is involved in the regulation of apoptosis and cell proliferation [36]. We unveiled a cadre of novel miRNAs that exhibited significant modulation in mice afflicted with colitis. Notably, miR-785-3p stood out as being prominently upregulated in colitis-afflicted mice. Prior research has elucidated that miR-785-3p exerts a suppressive influence on cell proliferation, migration, and invasion [37]. Furthermore, miR-335 was identified as another miRNA that experienced altered expression levels, with its downregulation associated with a reduction in pro-inflammatory gene expression triggered by α-synuclein, thereby ameliorating chronic neuroinflammation [38]. MiR-1260b was a kindly of miRNA that plays an important role in cancer by regulating Wnt signaling [39], which was also found to be regulated by DSS treatment. Therefore, differentially expressed miRNAs might extract potential targets that are involved in IBD.

Furthermore, we demonstrated that the target genes of miRNAs in the context of IBD exhibit a notable enrichment in immune response pathways. These encompass pivotal processes such as the positive regulation of the MAPK cascade, the positive regulation of NF-kappa B transcription factor activity, and the positive regulation of phosphatidylinositol 3-kinase signaling. In addition to immune-related pathways, our analysis unveiled the involvement of pathways linked to barrier function, notably encompassing cell junctions, adhesion junctions, and focal adhesions, in the intricate regulation orchestrated by miRNAs in IBD. Crucially, the differential expression patterns of these miRNAs in the context of IBD serve as indicators of phenotypic changes and are closely intertwined with the trajectory of disease evolution. These compelling findings collectively underscore the potential utility of miRNA profiling as a promising biomarker for both IBD diagnosis and prognosis.

Recent evidence indicates that disturbances in the gut microbiota and changes in miRNA expression may contribute to the development of IBD [15]. In a groundbreaking study, Liu et al. provided crucial insights into this topic by demonstrating the crucial role of host miRNAs in regulating intestinal homeostasis and shaping gut microbiota [14]. Certain miRNAs have been identified to directly regulate bacterial gene transcripts, thus promoting bacterial growth and motility and shaping the composition and distribution of the gut microbiota [40,41]. In addition, an increasing number of studies have investigated miRNAs as part of the immune response to bacteria, such as *Porphyromonas gingivalis*, which stimulates the upregulation of miR-146a expression and contributes to the elevated secretion of inflammatory cytokines, such as IL-1β, IL-6, and TNF-α [42]. These insights underscore the intricate and dynamic interplay between miRNAs, the gut microbiota, and the immune response, providing a deeper understanding of their collective role in the complex pathogenesis of IBD.

Within our study, we contribute additional compelling evidence to underscore the potential impact of the gut microbiota on host colonic miRNA expression. Specifically, we identified a selection of miRNAs, including miR-99b-5p, miR-93-5p, miR-503-5p, and miR-429-3p, that exhibited correlations with microbiota composition. Additionally, we made an intriguing observation of a negative association between *E. coli* and miR-200b-3p, which aligns with previous research demonstrating that miR-200b-3p possesses the capability to decrease *E. coli* levels [43]. As discussed, host miRNAs can shape the intestinal microbiota, and conversely, the gut microbiota can regulate miRNA expression, potentially leading to imbalances and diseases [15,44]. These results suggest that intestinal miRNA-microbiota interactions may be associated with inflammatory bowel disease pathophysiology. Future research should focus on targeting specific miRNAs or gut microbiota components as potential therapeutic strategies for IBD.

## 5. Conclusion

**Figure 8.**
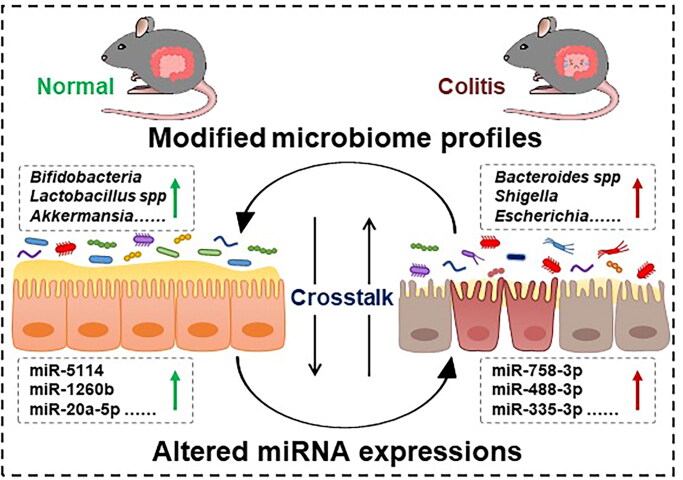
Complex crosstalk of intestinal microbiota and microRNA in colitis. The intersection between colitis, microbiota, and miRNA represent the points of influence between the disease and the profiles of specific intestinal bacteria and altered miRNA.

In conclusion, we identified 986 miRNAs between the two groups, with 41 upregulated and 21 downregulated miRNAs in colitis mice compared to the control group. Notably, the target genes of these significant miRNAs were primarily enriched in immune and inflammation-related pathways. Lefse analysis revealed bacterial biomarkers distinguishing the two groups, with notably higher levels of commonly encountered pathogens such as *Escherichia coli* and *Shigella flexneri* in the UC group, whereas beneficial species such as *Bifidobacterium pseudolongum* were more abundant in the control group. Metabolite analysis identified 3 downregulated and 20 upregulated metabolites in colitis mice. Spearman correlation further revealed the potential crosstalk between the microbiota profile and miRNAs in colitis (Figure 8). Understanding the interplay between gut microbiota and miRNAs may offer potential diagnostic and therapeutic targets for colitis.

## Supplementary Material

Supplemental Material

## Data Availability

The sequencing data were submitted to the NCBI SRA database using PRJNA795271 and PRJNA795830. The data supporting the findings of this study are available from the corresponding author upon request.
