# Supplementary material for: Multi-omics analysis reveals the interaction of gut microbiome and host microRNAs in ulcerative colitis
Source: Ann Med. 2023 Sep 29;55(2):2261477. doi: 10.1080/07853890.2023.2261477 (PMC10543339; doi:10.1080/07853890.2023.2261477)
Supplement: Supplemental Material [file IANN_A_2261477_SM0627.docx]

**Supporting information for**

**Multi-omics analysis reveals the interaction of gut microbiome and host microRNAs in Ulcerative Colitis**

**Supplementary Figure 1.**

**
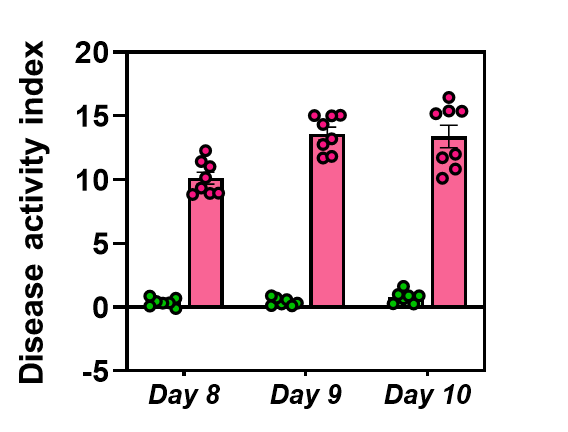
**

**Figure S1.** Disease activity index (DAI score) among two groups. DAI score are calculated as total score (body weight decrease + stool consistency + rectal bleeding) divided by 3.

**Supplementary Table 1. Primer sequence used for RT-PCR**

| Primer | | Forward | Reverse |
| --- | --- | --- | --- |
| mF4/80 | GCTGTGAGATTGTGGAAGCA | | CTGTACCCACATGGCTGATG |
| mCd206 | CAAGGAAGGTTGGCATTTGT | | CCTTTCAGTCCTTTGCAAGC |
| mCd11c | AAAATCTCCAACCCATGCTG | | CACCACCAGGGTCTTCAAGT |
| mT-bet | ATTCTCCACACCCTGTTTCG | | GATTCCTGGAAGGTGGTCAA |
| mGata3 | CCAATCCCAGTGACCTTGCT | | CTCGTCTCCAGGATGATGGC |
| mFoxp3 | GGTGCAGTCTCTGGAACAAC | | GGTGCCAGTGGCTACAATAC |
| mMCP1 | AGGTCCCTGTCATGCTTCTGG | | CTGCTGCTGGTGATCCTCTTG |
| mCcl5 | TGCCCTCACCATCATCCTCACT | | GGCGGTTCCTTCGAGTGACA |
| mβ-actin | AGGCCCAGAGCAAGAGAGGTA | | GGGGTGTTGAAGGTCTCAAACA |

**Supplementary Table 2 The number of modularity communities.**

| **Group** | **Nodes** | **Edges** | **Number of Communities** | **Module** | | | | | | | |
| --- | --- | --- | --- | --- | --- | --- | --- | --- | --- | --- | --- |
|  |  |  |  | **Ⅰ** | **Ⅱ** | **Ⅲ** | **Ⅳ** | **Ⅴ** | **Ⅵ** | **Ⅶ** | **Ⅷ** |
| Control | 185 | 1541 | 8 | 42 | 36 | 27 | 23 | 22 | 18 | 12 | 5 |
| Colitis | 195 | 1641 | 7 | 51 | 33 | 29 | 25 | 21 | 21 | 15 | - |
